# Secretory carcinoma of the breast containing the ETV6-NTRK3 fusion gene in a male: case report and review of the literature

**DOI:** 10.1186/1477-7819-3-35

**Published:** 2005-06-17

**Authors:** C Arce, D Cortes-Padilla, DG Huntsman, MA Miller, A Dueñnas-Gonzalez, A Alvarado, V Pérez, D Gallardo-Rincón, F Lara-Medina

**Affiliations:** 1 Division of Internal Medicine, Instituto Nacional de Cancerología, Mexico; 2 Division of Clinical Research, Instituto Nacional de Cancerología, Mexico; 3 Division of Pathology, Instituto Nacional de Cancerología, Mexico; 4 Unidad de Investigacion Biomédica en Cancer, Instituto de Investigaciones Biomedicas, Universidad Nacional Autonoma de Mexico e Instituto Nacional de Cancerología, Mexico; 5 Genetic Pathology Evaluation Center of the Departments of Pathology, British Columbia Cancer Agency Vancouver Canada; 6 General Hospital and University of British Columbia and the Prostate Centre at the Vancouver General Hospital, Vancouver, British Columbia, Canada

## Abstract

**Background:**

Secretory carcinoma (SC) of the breast is a rare and indolent tumor. Although originally described in children, it is now known to occur in adults of both sexes. Recently, the tumor was associated with the ETV6-NTRK3 gene translocation.

**Case presentation:**

A 52-year-old male was diagnosed with secretory breast carcinoma and underwent a modified radical mastectomy. At 18 months the tumor recurred at the chest wall and the patient developed lung metastases. He was treated concurrently with radiation and chemotherapy without response. His tumor showed the ETV6-NTRK3 translocation as demonstrated by fluorescent in situ hybridization (FISH).

**Conclusion:**

SC is a rare slow-growing tumor best treated surgically. There are insufficient data to support the use of adjuvant radiation or chemotherapy. Its association with the ETV6-NTRK3 fusion gene gives some clues for the better understanding of this neoplasm and eventually, the development of specific therapies.

## Background

SC of the breast is one of the rarest types of breast cancer accounting for less than 1% of all breast cancers. This entity was initially termed "Juvenile breast cancer" by McDivitt and Stewart, based on the fact that the average age of the seven patients described in their series was nine year-old with range of three to fifteen years [1]. Subsequently, more cases in children [2-6] and adults [7-12] were described. Therefore, it was recommended that the descriptive term SC replace the designation "juvenile carcinoma". Secretory breast carcinomas have a characteristic balanced translocation, t(12;15), that creates a ETV6-NTRK3 gene fusion. The finding of a fusion transcript in SC and the demonstration that ETV6-NTRK3 could transform murine mammary epithelial cell lines has challenged widely accepted beliefs on breast carcinogenesis [13]. This specific translocation is associated with congenital fibrosarcoma and mesoblastic nephroma, two morphologically similar pediatric mesenchymal tumors with no epithelial features [14]. The biological consequence of this translocation is the fusion of the dimerization domain of a transcriptional regulator (ETV6) with a membrane receptor tyrosine kinase (NTRK3) that activates the Ras-Mek1 and PI3K-Akt pathways which are important for breast cell proliferation and survival [15,17]. In only a few cases of secretory carcinoma the presence of the translocation has been confirmed. In the seminal report by Tognon 12 out of 13 cases tested positive [13] whereas Makretsov et al., found 3 out of 4 confirmed secretory carcinomas cases positive and at the same time screened 481 invasive breast carcinomas of which only one gave positive signal. This tumor was later confirmed to be a secretory carcinoma [16].

### Case report

A 52 year-old male presented to our institution having undergone local excision of a left breast tumor one month previously. The tumor had measured 7 × 5 cm. The mass had been present for 10 years. At physical examination there was evidence of recent surgery and the patient had a 1 cm ipsilateral axillary lymph node. Serum tumor markers and other routine blood test were normal. The liver ultrasonography, chest X-ray and bone scan were negative for metastases.

**Figure 1:**
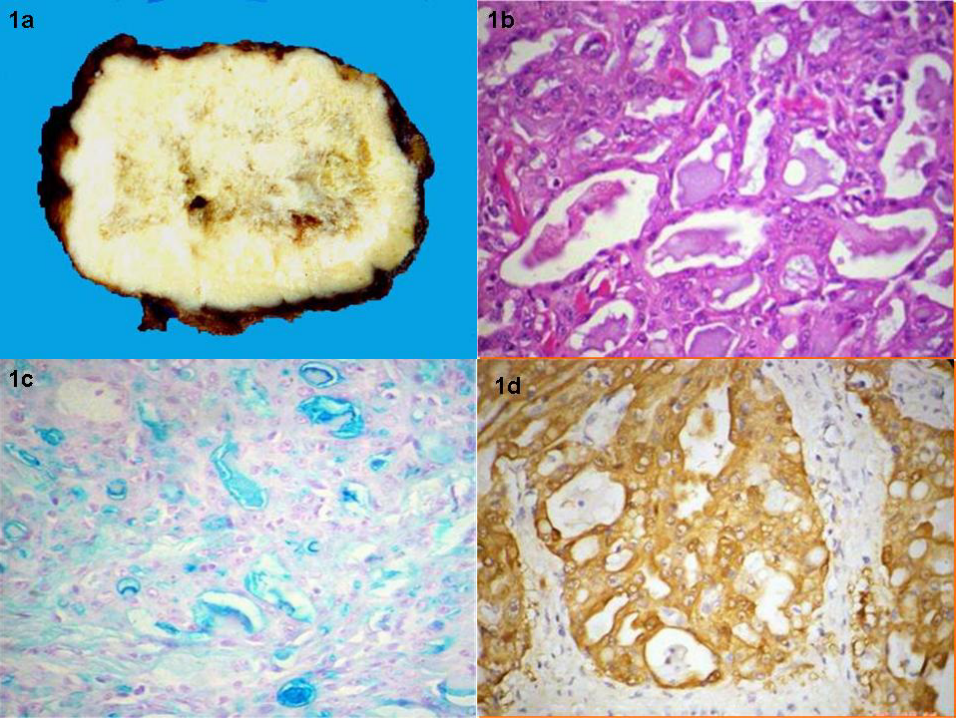
Tumor was grossly firm and circumscribed (1a). The histological pattern was microcystic (1b) with abundant intra and extracellular secretory material as showed by the colloidal iron stain which was diffuse and strongly positive (1c). The tumor was positive for S-100 protein (1d).

He underwent a modified radical mastectomy. Residual tumor measuring 2.8 cm × 2.6 cm was present. On macroscopic examination the tumor was firm and circumscribed (Figure 1a). Microscopy showed the classical features of secretory carcinoma with a microcystic pattern (Figure 1b) with abundant intra and extracellular secretory material. No tumor was present at the surgical margins. Colloidal iron staining highlighted the secretory material (Figure 1c). On immunohistochemistry, the tumor cells were positive for S-100 protein (Figure 1d) but negative for estrogen and progesterone receptor and HER2 (Dako, Carpinteria, CA). 2 of 24 resected lymph nodes were positive for metastatic carcinoma.

**Figure 2:**
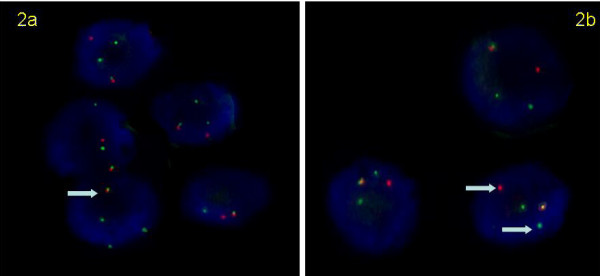
FISH images confirming the presence of the t(12;15). In 2a, the presence of the ETV6-NTRK3 fusion is demonstrated by the close proximity of a red signal (ETV6 from chromosome 12) with a green signal (NTRK3 from chromosome 15) in each cell. In 2b, each cell shows separation of red and green probes flanking the NTRK3 gene from chromosome 15.

The case was investigated for the t(12;15) ETV6-NTRK3 translocation using two complementary probe sets [16]. A t(12;15) translocation fusion probe assay (Fig. 2a) and a chromosome 15 NTRK3 gene split-apart assay (Fig. 2b) were used to detect the t(12;15) translocation. All BAC clones used in this study were obtained from the BACPAC Resources Centre at the Children's Hospital Oakland Research Institute. All probes were labeled by nick translation with the use of the manufacturer's recommended protocol (Vysis, Downer's Grove, Illinois). BAC clones RP11-434C1 and RP11-407P10 telomeric to ETV6 on 12p were labeled with spectrum orange. On chromosome 15, RP11-114I9 and RP11-730G13, centromeric to NTRK3 on 15q were labeled with spectrum green and clone RP11-247E14, telomeric to NTRK3 was labeled with spectrum orange. Six-micrometer tissue sections were baked overnight at 60C and then subjected to FISH with a modified protocol (Vysis, Downers Grove, IL) [14]. FISH signals were analyzed with a Zeiss Axioplan fluorescent microscope equipped with a COHU-CCD camera. Images were captures with Metasystems ISIS software (MetaSystems Group Inc., Belmont MA) with seven focal planes stacked for the analysis.

**Figure 3:**
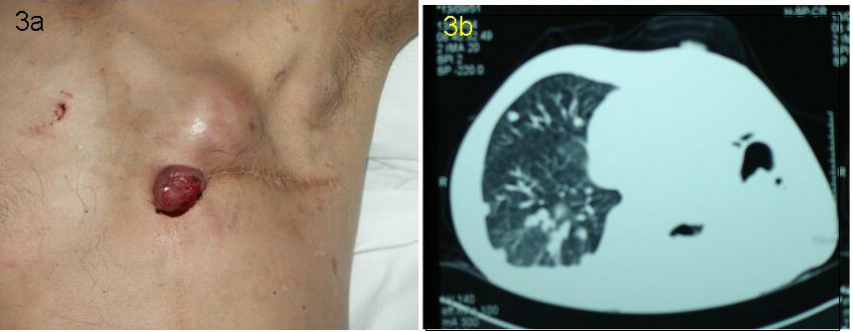
Disease recurrence at the chest wall (3a) and lung metastasis (3b). There were two hard nodules in the chest wall and three ipsilateral axillary nodules. In the lung several nodular metastases were present as well as a right sided pleural effusion.

In view of the nodal metastasis it was decided to treat the patient with six courses of adjuvant 5-fluorouracil, adriamycin and cyclophosphamide (FAC). The patient abandoned treatment after the second course. The patient returned to clinic eighteen months later with two hard nodules in the surgical resection area measuring 8 × 8 cm and 4 cm × 4 cm, (one ulcerated), and three left axillary subcutaneous nodules, two measuring 2 × 2 cm and one 3 × 3 cm (Figure 3a).

**Figure 4:**
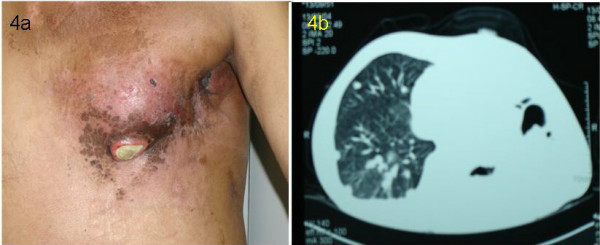
Disease in the chest wall (4a) and lung (4b) after radiation and concurrent chemotherapy. There was only minor response in the chest wall disease and essentially no change in the lung.

A chest CT scan identified pulmonary metastases with a right pleural effusion (Fig. 3b). The effusion was drained via percutaneous thoracentesis. He then began treatment with concurrent radiation (total dose of 60 Gy) and UFT (Tegafur-Uracil) to the chest followed by systemic UFT as a single agent for 3 months. Post-treatment, there was no change in the pulmonary disease and there was minor response of chest-wall and axillary disease (Figure 4a and 4b).

## Discussion

Secretory carcinoma is a very rare type of breast carcinoma. Lamovec and Bracko [18] reported 4 cases of SC in a retrospective series of 7038 breast carcinoma cases, and Botta et al [19] found one case of SC among 3000 breast carcinoma cases.

The age at presentation varies from 3 to 87 years with a median age of 25 years [1,5-11]. The male-female ratio is approximately 1:6 [20,21]. The case presented herein is extremely unusual as SC, particularly metastatic SC, has rarely been reported in males. Literature search identified only 16 other cases of SC in males. Our case was older than the average age reported for secretory carcinoma in males which is 17 years. Only the case reported by Kuwabara was older than our case (66 years) [22,23]. Table 1 summarizes the main clinical features of the cases of SC reported in males.

**Table 1:** Data on 17 males with Secretory Breast Cancer

Author	Year	Age	Duration of symptoms	Size (cm)	Axillary status	Treatment	Hormone Receptors	ETV6-NTRK3	Follow-up
Simpson^31^	1969	5	ND	ND	- (clinical)	LE	NE	NE	NED 4y
Tavassolli^7^	1980	9	ND	ND	- (clinical)	LE	NE	NE	NED 1.75y
Kari^5^	1985	3	1 mo	1.5	+ (1/4)	SM+ALNS	NE	NE	ND
Roth^32^	1988	23	21y	2.0	- (0/21)	MRM	NE	NE	NED 4y
Krausz^10^	1989	24	Many years	4.0	ND	SM + RT (axilla)	NE	NE	DOD 20y
Serour^21^	1992	17	4 y	1.5	- (0/3)	WLE + ALND	ER- PR+	NE	NED 5y
Lamovec^18^	1994	20	ND	1.2	- (0/?)	MRM	ER+ PR+	NE	NED 1y
Pohar-Marinsek^33^	1994	20	6–7 y	1.2	- (clinical)	SM	ER+ PR+	NE	NED 6 m
Kuwabara^34^	1988	66	3 y	3.0	+ (2/?)	MRM	ER- PR+	NE	NED 8 m
Vesoulis^35^	1998	33	10 y	1.5	ND	MRM	ER+ PR+	NE	ND
Kameyama^36^	1998	50	ND	3.0	- (0/?)	MRM	ER+	NE	ND
Chevallier^37^	1999	9	14 m	2.0	- (0/?)	LE + ALND	ER- PR-	NE	NED 45 m
Yildirim^38^	1999	11	1 y	1.5	+ (1/18)	MRT + CT+ RT	ER -	NE	NED 12 m
Bhagwandeen^39^	1999–2000	9	1 m	1.2	- (0/15)	MRM	ER- PR-	NE	NED 20 m
De Bree^22^	2001	17	2 y	2.0	- (0–14)	MRM	ER- PR-	NE	NED 9 m
Grabellus^40^	2005	46 Male-female transexual	ND	4.0	ND	LE	ER- PR-	PRESENT	ND
This case	2005	52	10 y	7	+ 2/24	MRM + CT	ER- PR-	PRESENT	AWD 25 m

ND: no defined, LE: Local excision, MRM: Modified Radical Mastectomy, CT: chemotherapy, RT Radiotherapy, NE: not examined, NED: not evidence of disease, AWD: Alive with disease, ER: estrogen receptor, PR progesterone receptor. SM simple mastectomy, ALNS: axillary lymph node sampling, ALND: axillary lymph node dissection, WLE: wide local excision, DOD: Died of disease.

SC's can demonstrate several histological patterns including, solid, microcystic, and ductal, with many tumors containing all three patterns [24]. The tumor cells are polygonal with granular eosinophilic cytoplasm. Atypia is minimal or absent and mitotic activity is low [20]. A typical finding is the presence of intracellular and extracellular secretions [7]. This secretory material is periodic acid-Schiff and alcian blue positive [24,25]. In this tumor there was no expression of steroid receptors or HER2. A recent study has reported than only 4 and 2 out of 13 cases expressed the estrogen and progesterone receptor respectively and only two were HER2 positive [26]. In the current case, the tumor had the t(12;15) ETV6-NTRK3 fusion gene (Fig. 4).

The most frequent clinical presentation is of an asymptomatic mobile mass, which is usually subareolar. The tumor size varies from 1 cm to 16 cm with an average diameter of 3 cm. [7,16]. Our patient had a mass of 7 cm × 5 cm. As the patient reported that the lesion had been present for at least 10 years, it had behaved in a slow growing, indolent fashion. This is supported by other reported cases [20]. In this regard, Biallo et al., have reported a MIB1 labeling index of 11.4% (range: <1 to 34%) [26].

Surgery is considered the primary treatment of secretory carcinoma, however, due to scarcity of reported cases no published guidelines for surgical management exist. There are however, many cases reported of patients who had suffered a local recurrence, therefore mastectomy appears to be a sound surgical choice [1,5,7,9-11,22,24,25]. There are no data however on conservative treatment but this option could be explored particularly in cases where breast development has not yet occurred. In regard to the management of the axilla, the overall incidence of axillary lymph node infiltration is around 30% in children and adults regardless of gender [21,24], hence axillary lymph node dissection is advocated by some authors for tumors ≥2 cm [7,24]. Nevertheless, sentinel node biopsy, may be useful for secretory carcinomas. A recent report on a 9-year-old girl treated with simple mastectomy and axillary sentinel lymph node biopsy shows that this is feasible [27].

Postoperative radiotherapy [19,21] and adjuvant chemotherapy [7,28] have been used on at least two occasions. There is at present insufficient evidence to recommend either approach in the management of secretory carcinoma.

Local recurrence after a long disease-free interval has been described in numerous cases; [1-3,5,9,11,28] however these occurred in patients that underwent conservative surgery. This is the second case reported with chest wall recurrence after mastectomy. In the other case the patient was treated with wide local excision and she is alive at 11-month follow-up. In contrast, our case also presented distant recurrence [12].

Distant metastases from secretory carcinoma are extremely rare with only four cases reported [20]. Our case is the fifth case who developed distant metastases, this, despite having only two positive lymph nodes at resection. Another recently reported patient remained disease free at a follow-up of 13 months despite having 12 out of 14 positive nodes and not having received adjuvant chemotherapy [23].

There are several reported cases of patients with secretory breast carcinoma with distant metastases who were treated with either single agent or combination chemotherapy without success. Among the drugs reported are 5-FU, vindesine, mitomycin and prednisone, adriamycin, epirubicin, cyclophosphamide, carboplatin, and even newer active agents such as docetaxel. These data clearly show that this neoplasm is not chemosensitive, as all of the patients treated with chemotherapy showed disease progression while on treatment [7,10,20] and [29]. In our case, despite using UFT alone and with concomitant radiotherapy there was no response. These observations are in contrast with reports on the high chemosensitivity to common agents (vincristine, cyclophosphamide, adriamycin, dactinomycin and ifosfamide) for congenital fibrosarcomas and mesoblastic nephromas, two other neoplasms associated with the translocation ETV6-NTRK3 [30]. This suggests that secretory breast carcinoma, due perhaps to its slow growing behavior, acquires additional genetic alterations than ultimately confer chemoresistance. It will be very useful to establish cancer cell lines from this tumor type to study whether the chemoresistance is a general phenomenon or drug specific.

## Conclusion

Secretory carcinoma is a rare slow-growing tumor that is best approached by surgical treatment. There are insufficient data to support the use of adjuvant radiation and/or chemotherapy. Its association to the ETV6-NTRK3 fusion gene gives some clues for the better understanding of this neoplasm and may eventually lead to the development of specific therapies.

## Competing interests

The author(s) declare that they have no competing interests.

## Authors' contributions

C A-S, and D C-P conceived the study and wrote the manuscript; DG H and MA M performed the FISH analysis and participated in the discussion and writing of the manuscript; V P-S, did the pathological analysis; A A, and F L-M cared for the patient; D G-R critically reviewed the manuscript; AD-G participated in the discussion and writing of the manuscript.
